# TRODAT SPECT in patients with idiopathic REM sleep behaviour disorder

**DOI:** 10.5935/1984-0063.20180014

**Published:** 2018

**Authors:** Geraldo Nunes Vieira Rizzo

**Affiliations:** 1 Hospital Moinhos de Vento, Sonolab - Porto Alegre - RS - Brasil.

**Keywords:** Single Photon Emission Computed Tomography Computed Tomography, REM Sleep Behavior Disorder, Parkinson Disease

## Abstract

to determine the value of dopamine transporter (DAT) neuroimaging radiotracer in a group of idiopathic rapid eye movement sleep behavior disorder (iRBD) patients regarding the development of a synucleinopathy. **Methods:** 6 retrospectively selected patients with clinical and polysomnographic diagnosis of iRBD, on treatment or not, were submitted to a single-photon emission computerized tomography (SPECT) with TRODAT. **Results:** Five from six patients have an abnormal test showing reduction in DAT density measured by the linkage potential on SPECT. **Conclusions:** TRODAT crosses the blood brain barrier, has a high affinity for DAT and is capture by SPECT. The decreased uptake of DAT tracers means a reduction in dopaminergic activity which suggest the possibility of Parkinson Disease. We have tried to reinforce iRBD as a marker of neurodegenerative disease and suggest SPECT with TRODAT as an easy method in our country to follow longitudinally these patients.

## INTRODUCTION

In the last decades, the importance of nonmotor manifestation of Parkinson’s Disease (PD) is clearly being considered a prodromal stage of the diseases^1^-^2^. Rapid eye movement sleep behavior disorder (RBD) is a well-known parasomnia, disturbance characterized by loss of muscle atonia during REM sleep and dream-enacted behaviors, with most of the dreams being violent or aggressive, so that patients often come to see the doctor complaining hurting themselves or bed partners during sleep.

Prevalence of RBD based on populations is less than 3% but it is much higher in old people and in those suffering from a neurodegenerative disease. Available data indicate that most of the patients with the idiopathic form of the disease (iRBD) develop a synucleinopathy, mainly Parkinson’s Disease (PD) and dementia with Lewy bodies (DLB)^3^-^6^. Several important cohort studies on iRBD patients were done by Schenck et al., in 1996/2013, Iranzo et al., in 2006/2013 and Postuma et al., in 2009^3^-^6^. However, the time lag between diagnosis of iRBD and synucleinopathy is quite variable, the risk of neurodegenerative disease increases with the duration of iRBD and there is no neuroprotective or disease-modifying drug yet. Since we now consider iRBD a marker of neurodegeneration we expect, in the near future, to easily and as soon as possible to identify these patients to stop the underlying neurodegenerative process.

**Figure 1:**
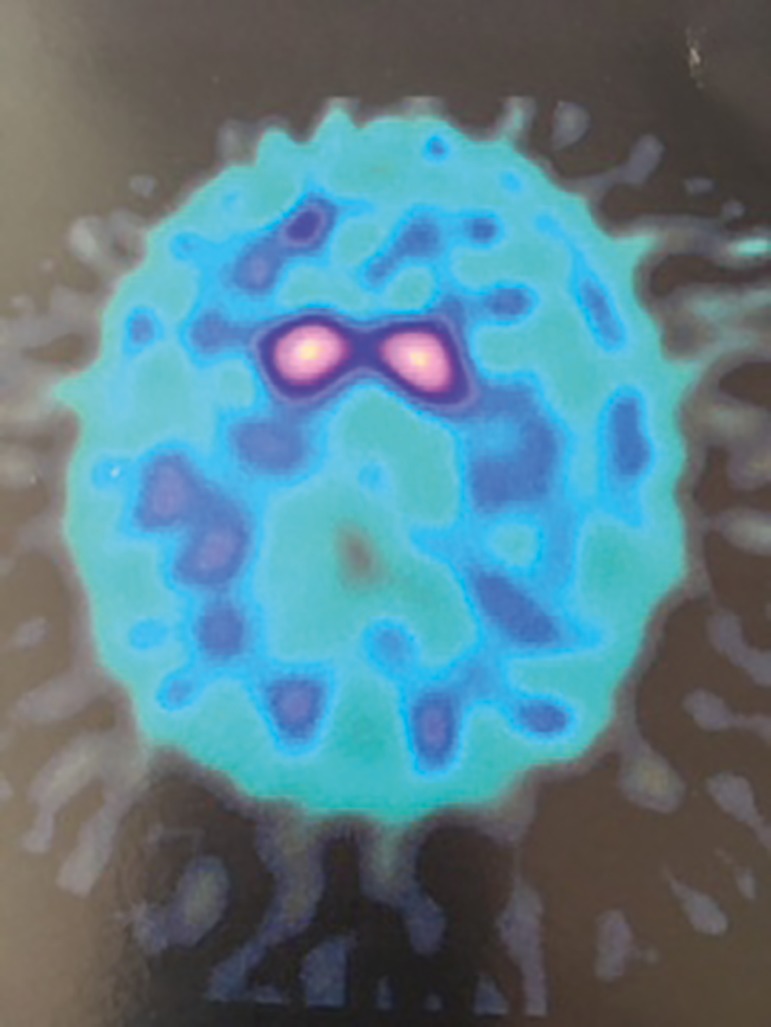
Abnormal exam.

**Figure 2:**
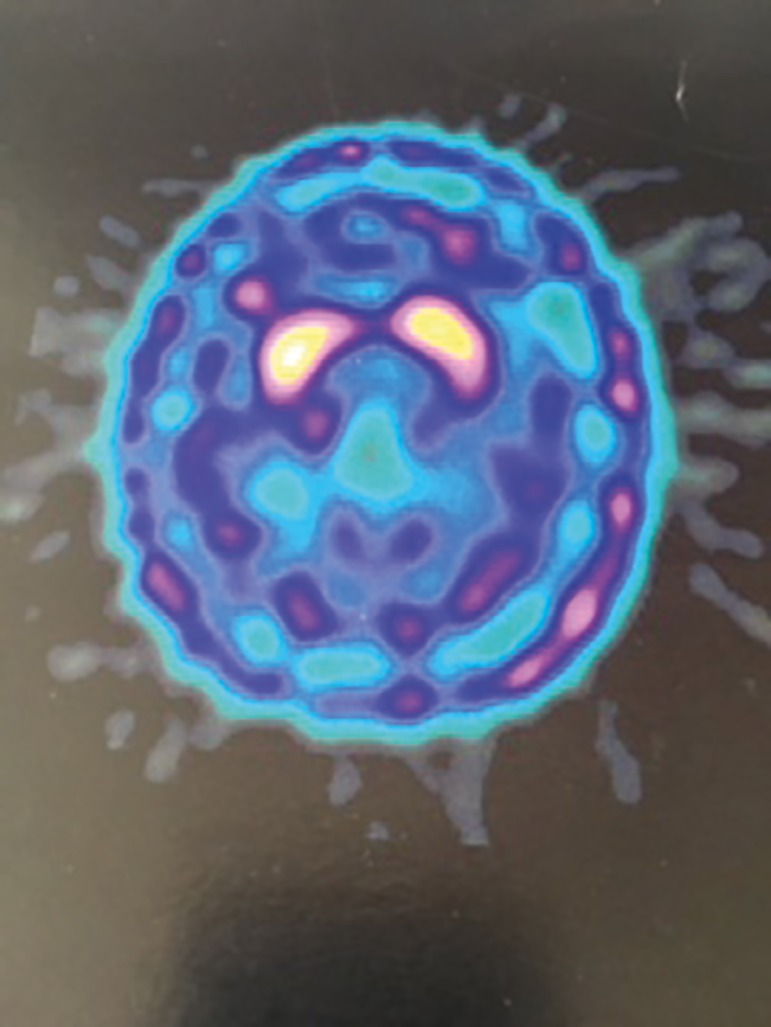
Normal exam.

The imaging of dopamine transporter (DAT) with (99m) Tc-TRODAT-1 and SPECT has been proposed to be a valuable and feasible method to evaluate the integrity of dopamine neurons.^7^-^8^ TRODAT is a marker that selectively binds to the presynaptic dopamine receptors present in our brain. The loss of dopamine receptors is associated with the progression of PD (fig 1) and, in turn, normal TRODAT SPECT images (fig 2) rule out the hypothesis of the disease.

SPECT with TRODAT offers great advantages compared to other tracers because it presents the same efficiency with much lower cost. The other tracers use isotopes such as [123I) for SPECT and [11C] and [18F] for positron emission tomography (PET)have little availability in Brazil and cost, at least, 10 times more when compared to 99mTc

## MATERIAL AND METHODS

Fifty-eight patients with polysomnography (PSG) confirming RBD were retrospectively selected from 2012-2016. From this group 39 were excluded due to parkinsonian signs and/or symptoms and 10 in use of antidepressants. We then proposed to this group of 9 patients the realization of SPECT with TRODAT and only 6 have agreed with the procedure. At the time of neuroimaging, all 6 patients were free of neurological diseases, were not using psychoactive drugs, and the neurologic evaluation exclude the presence of parkinsonism, mild cognitive impairment (MCI) or even dementia.

Our sample was constituted of 6 male patients with age varying between 62-81 y.o. and diagnosed with idiopathic REM Behavior Disorder (iRBD) following ICSD-3 criteria. Being treated was not considered an exclusion factor. The time interval between the first symptoms and the diagnosis has varied from 1-5 years.

We do not have a control group.

The images were obtained 4 hours after the intravenous injection of the tracer. The ratios of specific striatal binding to nonspecific occipital binding were calculated (Table 1). The linkage potential (LP) considered as a reference for normality in our Hospital is more than 1,1.

**Table 1:** Results.

	First symptoms	RBD Diagnosis	TRODAT	Left CP	Right CP
Subject 1 male 62 y.o	2008	2012	2016	0.20	0.44
Subject 2 male 72 y.o	2012	2014	2016	0.54	0.54
Subject 3 male 71 y.o	2009	2012	2016	0.77	0.50
Subject 4 male 67 y.o	2013	2016	2016	0.75	1.05
Subject 5 male 81 y.o	2014	2015	2016	0.48	0.52
Subject 6 male 63 y.o	2010	2015	2016	1.23	1.11

## RESULTS

Five out of 6 patients (84%) with iRBD examined with TRODAT SPECT have abnormal exam (Fig. 2) with bilateral uptake reduction of DAT tracer in the striatum (Table 1).

## DISCUSSION

A history of recurrent nocturnal dream enactment behaviors (DEBs) plus a Polysomnography (PSG) finding of REM Sleep muscle atonia leads us to the diagnosis of RBD. The degeneration of the locus coeruleus/subcoeruleus complex is supposed to be the cause of RBD and clonazepam is the most used drug to treat it. Lately an enormous number of articles have been written regarding RBD due to its increased association with neurodegenerative diseases, mainly synucleinopathies caused by the pathologically deposition of alpha-synuclein, including Parkinson’s Disease (PD), Multiple System Atrophy (MSA) and Dementia of Lewy Bodies (DLB).

In each of these studies is there allways a highlight for the interval from RBD diagnosis to clinical signs and symptoms of a neurogenerative disease in order to figure out the risk of such disease. The most important studies on this field are those of Schenck et al., Iranzo et al. and Postuma et al., but there is an international group of scholars dedicated to RBD and a lot of research going on. Their hope is to find a protective drug to avoid this evolution.

For decades, it has been possible to measure dopaminergic innervation using PET and SPECT and normal scans are a strong sign against DP being an exclusion criterion from probable PD diagnosis according to International Movement Disorders Society^7^. The same society now admits that iRBD represents a prodromal PD^1^.

The reduction of DAT density occurs even before the onset of PD symptoms, since there is a 40 to 60% reduction in dopaminergic activity (uptake of DAT tracers) when the first symptoms appear and, with the evolution of the disease, the levels of uptake decrease by up to 90%^9^-^11^. It is for this reason that the concentration of DAT in the evaluation of the loss of dopaminergic neurons in the striatum, more specifically in the putamen, has been shown to be a useful parameter both in the early diagnosis of PD and in the differential diagnosis with other diseases that induce extrapyramidal signs or symptoms.

Knowing that iRBD is a marker of neurodegeneration specially regarding to synucleinopathy like PD and DLB we have tried to reinforce this with a feasible method. Clearly dopaminergic functional imaging will become a key part of the future of prodromal PD. So, we can follow these patients while we hope the pharmaceutical industry can find a drug to stop the neurodegenerative process.

In Brazil, the scintigraphy with marker of DAT is done with the radioisotope TRODAT-Tc99 which is able to differentiate forma of degenerative parkinsonism from other conditions like essential tremor, drug induced parkinsonism e psychogenic parkinsonism^12^. However this method is not able to differentiate idiopathic PD from other types of degenerative parkinsonism like multiple system atrophy (MSA) and progressive supranuclear paralysis (PSP).

A recent study of 35 iRBD patients who underwent DAT-SPECT concluded those with decreased TRODAT binding in the left putamen had a relatively higher risk of developing neurodegenerative synucleinopathy disease after a median of 4 years of prospective follow-up^13^.
